# “*Candidatus* Neoehrlichia mikurensis” in *Ixodes ricinus* ticks collected near the Arctic Circle in Norway

**DOI:** 10.1186/s13071-018-3168-y

**Published:** 2018-12-04

**Authors:** Clarinda Larsson, Dag Hvidsten, Snorre Stuen, Anna J. Henningsson, Peter Wilhelmsson

**Affiliations:** 1 Faculty of Medicine and Health Sciences, Linköping University, Linköping, Sweden; 2 Department of Microbiology and Infection Control, University Hospital of North Norway, Tromsø, Norway; 3 Department of Laboratory Medicine, Nordland Hospital, Bodø, Norway; 4 Department of Production Animal Clinical Sciences, Section of Small Ruminant Research and Herd Health, Norwegian University of Life Sciences, Sandnes, Norway; 5 Clinical Microbiology, Laboratory Medicine, County Hospital Ryhov, Jönköping, Sweden; 6 Department of Clinical and Experimental Medicine, Linköping University, Linköping, Sweden; 7 Division of Medical Microbiology, Department of Clinical and Experimental Medicine, Linköping University, Linköping, Sweden; 8 Department of Medical Microbiology, Laboratory Medicine, County Hospital Ryhov, Jönkoping, Sweden

**Keywords:** “*Candidatus* Neoehrlichia mikurensis”, *Ixodes ricinus*, Neoehrlichiosis, Tick-borne pathogen, Arctic Circle, Norway

## Abstract

**Background:**

“*Candidatus* Neoehrlichia mikurensis” is a gram-negative bacterium belonging to the family *Anaplasmataceae* that, in Europe, is transmitted by *Ixodes ricinus* ticks. “*Candidatus* N. mikurensis” can cause a severe systemic inflammatory syndrome, neoehrlichiosis, mostly in persons with other underlying diseases. To date, “*Ca.* N. mikurensis” has been found in ticks in different countries in Asia and Europe, but never as far north as at the Arctic Circle.

**Methods:**

A total of 1104 *I. ricinus* ticks collected from vegetation and from animals in northern Norway (64–68°N) were analysed for the prevalence of “*Ca.* N. mikurensis”. Of them, 495 ticks were collected from vegetation by flagging and 609 ticks were collected from dogs and cats. Total nucleic acid extracted from the ticks were converted to cDNA and analyzed with real-time PCR targeting the *16S* rRNA gene of “*Ca.* N. mikurensis”. Positive samples were further analysed by nested PCR and sequencing.

**Results:**

“*Candidatus* N. mikurensis” was detected in 11.2% of all collected *I. ricinus* ticks in northern Norway. The prevalence differed between ticks collected from vegetation (18.2%; 90/495) compared to ticks collected from dogs and cats (5.6%; 34/609). The ticks from dogs and cats were collected in Brønnøy area and seven additional districts further north. The prevalence of “*Ca.* N. mikurensis” in these ticks differed between geographical localities, with the highest prevalence in the Brønnøy area.

**Conclusions:**

The detection of “*Ca.* N. mikurensis” in *I. ricinus* ticks from the Arctic Circle in northern Norway indicates potential risk for tick-bitten humans at this latitude to be infected with “*Ca.* N. mikurensis”.

## Background

“*Candidatus* Neoehrlichia mikurensis” is a relatively recently discovered tick-borne pathogen that has been shown to cause a severe systemic inflammatory syndrome, neoehrlichiosis, mostly in persons with other underlying diseases. The first description of the bacterium was published as late as 1999 [1] and the first human cases were described in 2010 [2, 3].

“*Candidatus* N. mikurensis” is a small, obligately intracellular gram-negative coccus belonging to the family *Anaplasmataceae*, order Rickettsiales. It was described as a new genus for the first time (2004) when it was found in *Ixodes ovatus* ticks and isolated from brown rats (*Rattus norvegicus*) in the Japanese island Mikura [4]. Later, it turned out that the bacterium already had been detected in *Ixodes ricinus* ticks in the Netherlands, but at that time, it was just classified as ungrouped *Ehrlichia* DNA and named the “*Schotti-variant*” [1]. “*Candidatus* N. mikurensis” is widespread among *I. ricinus* ticks and rodents in Europe [5–7]. A compilation of studies from 16 European countries shows that the prevalence in ticks collected from vegetation ranges from below 1% to over 20%, whereas the prevalence in *I. ricinus* is, in on average, around 6–8% [8]. Earlier studies in Europe have shown that ticks with co-infection of “*Ca*. N. mikurensis” and *Borrelia afzelii* are more common than ticks having other co-infections with “*Ca.* N. mikurensis” [9].

Very little is known about “*Ca.* N. mikurensis” and its pathogenicity. It is only recently that the ability of “*Ca.* N. mikurensis” to cause serious disease in humans has become known. The first published human case of neoehrlichios was a 77 year-old man from Gothenburg, Sweden [3]. Currently there are more than a dozen cases of serious infection with “*Ca.* N. mikurensis” described in the literature. In most cases these are persons with underlying autoimmune or hemolytic diseases [2, 3, 8, 10] or people treated with cytostatic and immunosuppressive drugs [11]. The most common symptoms described are high and remitting fever; vascular and thromboembolic events are also common, as well as skin rashes [12].

There is reason to suspect that neoehrlichiosis is an underdiagnosed infection because most of the infected patients already have another disease and it is easy to overlook an infection with “*Ca.* N. mikurensis” as a cause of the patient’s symptoms. Even the difficulty of detecting the bacterium (it does not grow in ordinary culture media, serology is not yet available and molecular detection methods are only available in a few laboratories) probably contributes to the fact that it is a rare diagnosis.

The aim of the present study was to examine how far north it is possible to find *I. ricinus* ticks with “*Ca*. N. mikurensis” infection. The area around Brønnøy at the Arctic Circle in northern Norway has previously been shown to constitute the northern distribution limit for *I. ricinus* and in this study the prevalence of “*Ca.* N. mikurensis” was examined in ticks collected from the area. Two different materials of ticks were analysed; ticks collected from vegetation and ticks collected from dogs and cats. The ticks had previously been analysed for infections with *B. burgdorferi* (*s.l.*) [13, 14], and the occurrence of coinfected ticks was therefore investigated in the present study.

## Methods

### Tick collection

**Fig. 1:**
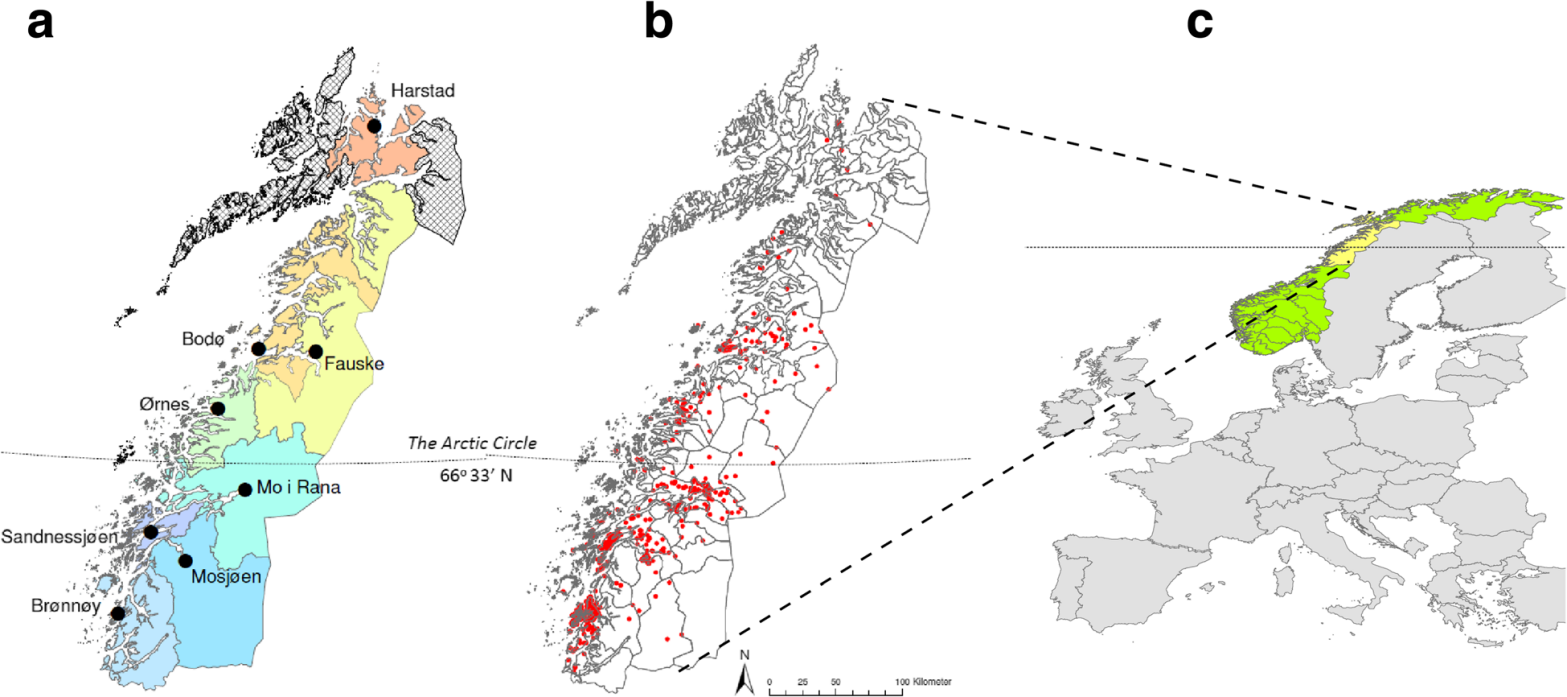
**a** The study area covers 8 districts in northern Norway (from 64°56'N–68°48'N) across the Arctic Circle (66°33'N). The Vesterålen and Lofoten archipelago (hatched area, left) and Narvik city (hatched area, right) were not included in the study. **b** Scatter diagram of the origin of ticks in the study area (red dot = one single tick). In the southernmost district (Brønnøy) 244 ticks were collected, and in the northernmost district (Harstad) 6 ticks. **c** Sketch map showing the study area in relation to western Europe. Copyright: Creative Commons Attribution 4.0 License (https://creativecommons.org/licenses/by-nc-nd/4.0/deed.no). Citation: Hvidsten et al. (2014) *Ixodes ricinus* and *Borrelia* prevalence at the Arctic Circle in Norway. *Ticks Tick Borne Diseases*. 2014;5:107–12 [14]

The method of collection and analysis of the ticks has been described previously [13, 14]. In brief, from April to November 2011, *I. ricinus* ticks were collected from vegetation by flagging, at two sites in Brønnøy in northern Norway [13]. In addition, *I. ricinus* ticks were collected from dogs and cats, at different veterinary stations in the Brønnøy area and seven additional districts further north during 2010 and 2011 (Fig. 1) [14]. Tick species and developmental stages were determined by using a stereomicroscope [15–17].

### Extraction of total nucleic acid and cDNA synthesis

The procedure for extraction of total nucleic acid and cDNA synthesis has been described previously [18]. In brief, a lysis buffer containing β-mercaptoethanol was added into the tubes that contained the ticks. Tubes were then frozen for 1 h at -180 °C, disruption with bead beating and centrifugation was performed, and the supernatant used for total nucleic acid extraction. Synthesis of cDNA was performed using the Illustra Ready-to-Go RT-PCR Beads kit (GE Healthcare Bio-Sciences AB, Stockholm, Sweden).

### Detection of “*Ca.* N. mikurensis” using real-time PCR

Detection of “*Ca*. N. mikurensis” was performed using a SYBR green real-time PCR assay, as previously described [19]. The primers NEO_16S_F and NEO_16S_R were designed to target the “*Ca*. N. mikurensis” *16S* rRNA gene, amplifying a 107 bp long amplicon (Table 1).

**Table 1:** Primers targeting the “*Ca.* N. mikurensis” *16S* rRNA gene used for screening and sequencing. Establishment of the primers has previously been described previously [19]

Primer	Sequence (5'-3')
Neo_16S_F	GTAAAGGGCATGTAGGCGGTTTAA
Neo_16S_R	TCCACTATCCTCTCTCGATCTCTAGTTTAA
Neo_16S_95_F	TTAGTGGCAGACGGGTGAGTAATG
Neo_16S_127_F	TCTGCCTAGTAGTATGGAATAGCTG
Neo_16S_1363_R	AAACCAATTTCCAGGGCATGACGG
Neo_16S_1393_R	TCCTTACGGTTAGCTCACCAGCTT

The 20 μl reactions consisted of 10 μl of Maxima SYBR Green mix (Life Technologies, Vilnius, Lithuania), 0.4 μl of each primer (10 μM, Invitrogen, Paisley, United Kingdom), 7.2 μl of RNAse-free water and 2 μl of template. PCR reactions were performed on a C1000^TM^ Thermal Cycler, CFX96^TM^ system (Bio-Rad Laboratories, Inc., Hercules, CA, USA) using an activation step at 95 °C for 3 min, and 45 cycles of 95 °C for 15 s, 60 °C for 30 s, and 72 °C for 30 s. Immediately after completion of the PCR cycles, melting curve analyses were performed by heating to 95 °C for 15 s, followed by cooling to 60 °C for 1 min, and subsequent heating to 95 °C at 0.8 °C/min with continuous fluorescence recording. As a positive control, cDNA samples positive for “*Ca*. N. mikurensis” confirmed by sequencing in an earlier study [19] were used in each run.

A sample was considered as positive for “*Ca.* N. mikurensis” when the melting temperature was 74.5 °C.

### Nested PCR assay and sequencing

In order to further validate samples positive for “*Ca*. N. mikurensis” in the SYBR green real-time PCR assay, a conventional nested PCR assay including primers targeting the “*Ca*. N. mikurensis” *16S* rRNA gene, to amplify a 1262 bp long amplicon, was used [19].

The 25 μl reactions consisted of 5 μl of 5 × Phusion^TM^ HF Buffer (Thermo Scientific, Vilnius, Lithuania), 1.25 μl of each of the primers Neo_16S_95_F and Neo_16S_1393_R (10 μM, Invitrogen, Table 1), 0.5 μl of dNTP mix (10 mM), 0.25 μl of Phusion^TM^ HF DNA polymerase (Thermo Scientific), 14.75 μl of RNAse-free water and 2 μl of template.

The PCR reactions were performed on a thermo block instrument (Corbett Research, Techtum Lab, Nacka, Sweden) using an activation step at 98 °C for 3 min, and 45 cycles of 98 °C for 30 s, 58 °C for 40 s and 72 °C for 60 s, with a final extension at 72 °C for 7 min.

An aliquot (5 μl, diluted 1:100 with RNAse-free water) of the PCR product obtained in this assay was added to a second PCR mixture, which was prepared using the same volumes and concentrations as used for the first mixture, except with a different primer pair (Neo_16S_127_F and Neo_16S_1363_R, Invitrogen, Table 1). The temperature cycles used in the nested PCR assay was 98 °C for 3 min, and 45 cycles of 98 °C for 30 s, 55 °C for 40 s and 72 °C for 60 s, with a final extension 72°C for 7 min.

GATC Biotech AG (Köln, Germany) performed nucleotide sequencing of the PCR products obtained from the nested PCR assay. Chromatograms were edited using BioEdit Software v7.0 (Tom Hall, Ibis Therapeutics, Carlsbad, CA, USA) and sequences were examined using the Basic Local Alignment Search Tool (BLAST).

### Statistics

Statistical analyses were performed with IBS software SPSS Statistics version 24. For comparison of “*Ca.* N. mikurensis” prevalence between subgroups of ticks [depending on geographical collection site, life stage, sex, and co-infection with *B. burgdorferi* (*s.l.*)], Pearson’s Chi-square test was used, and *P* < 0.05 was regarded as significant.

## Results

A total of 1104 ticks were analysed for the presence of “*Ca.* N. mikurensis”. Of them, 124 (11.2%) were positive for “*Ca.* N. mikurensis” using the real-time PCR assay (Table 2). Of these positive samples, 96 samples could be confirmed as positive after sequencing when compared to known “*Ca.* N. mikurensis” sequences. Because the analysed samples are derived from ticks with different origins and collected at different occasions, the results will hereafter be reported separately for ticks collected from dogs and cats and ticks collected from vegetation.

**Table 2:** The number of samples that has been analyzed for “*Ca.* N. mikurensis” and the proportion of positive samples in real-time PCR

	No. of negative samples	No. of positive samples (%)	Total number
Ticks collected April to November 2011 by flagging in Brønnøy [12]	405	90 (18.2)	495
Ticks collected from dogs and cats in Brønnøy and 7 additional districts during 2010–2011 [13]	575	34 (5.6)	609
Dogs	292	22 (7.0)	314
Cats	283	12 (4.1)	295
Total number	980	124 (11.2)	1104

### *Ixodes ricinus* ticks collected from dogs and cats

A total of 609 samples from *I. ricinus* ticks from dogs and cats were analyzed, 314 were from ticks found on dogs and 295 from ticks found on cats. Of these 609 samples, 5.6% (34/609) were positive for “*Ca.* N. mikurensis”. The percentage of positive samples was slightly higher among those from dogs, 7.0% (22/314), compared to 4.1% (12/295) of the ticks from cats, but the difference was not statistically significant (Chi-square test*: χ*^2^ = 2.492, *df* = 1, *P* = 0.114).

Most of the ticks (261/609) from dogs and cats were collected in the Brønnøy area and it was also in Brønnøy that the prevalence of “*Ca.* N. mikurensis”-infected ticks was highest, with 10.3% of samples positive. The higher prevalence in Brønnøy compared to all the other geographical areas taken together were significant (Chi-square test*: χ*^2^ = 22.685*, df* = 8, *P* = 0.004). After Brønnøy, ticks collected in Fauske had the second highest proportion of positive samples, 6.2% (2/32). “*Candidatus* N. mikurensis”-positive samples were also found in ticks collected in Sandnessjøen, Bodø and Mosjøen, but with a lower prevalence (Sandnessjøen, 4.6%, 3/65; Bodø, 1.9%, 1/52; and Mosjøen, 1.8%, 1/55). In ticks from dogs and cats collected in the other places in the study, all test results were negative (Table 3).

**Table 3:** The geographical distribution of ticks negative and positive for “*Ca.* N. mikurensis” collected by flagging in Brønnøy and from dogs and cats in Brønnøy and seven additional districts in northern Norway

	No. of negative samples	No. of positive samples	Positive samples (%)	Total number
Bodø	51	1	1.9	52
Brønnøy	234	27	10.3	261
Fauske	30	2	6.2	32
Harstad	6	0	0	6
Mosjøen	54	1	1.8	55
Mo i Rana	98	0	0	98
Sandnessjøen	62	3	4.6	65
VL^a^	3	0	0	3
Ørnes	37	0	0	37
Total number (dogs and cats)	575	34	5.6	609
Brønnøy (flagging)	405	90	18.2	495

^a^VL, Vesterålen Lofoten. The area is not marked in Fig. 1 but is located northwest of the map

Of the 609 *I. ricinus* ticks collected from dogs and cats, 84.6% (515/609) were adult females, 11.7% (71/609) were adult males, 0.7% (4/609) nymphs and 3.1% (19/609) ticks were of uncertain life stage and sex. The prevalence of “*Ca.* N. mikurensis” was lowest in adult females, 4.5% (23/515), followed by unidentified stages 10.5% (2/19) and then adult males 11.3% (8/71). Of the four nymphs, one was positive for “*Ca.* N. mikurensis” (Table 4).

**Table 4:** The distribution of “*Ca*. N. mikurensis”-positive samples between the various stages of *I. ricinus* ticks collected in northern Norway

			Female	Male	Nymph	Larva	Unidentified	Total
Dogs and cats	Negative	Count	492	63	3	0	17	575
	Positive	Count	23	8	1	0	2	34
		% within life stage and sex	4.5	11.3	25.0	–	10.5	5.6
Total		Count	515	71	4	0	19	609
Flagging	Negative	Count	53	68	253	31	0	405
	Positive	Count	13	11	65	1	0	90
		% within life stage and sex	19.7	13.9	20.4	3.1	0	18.2
Total		Count	66	79	318	32	0	495

**Fig. 2:**
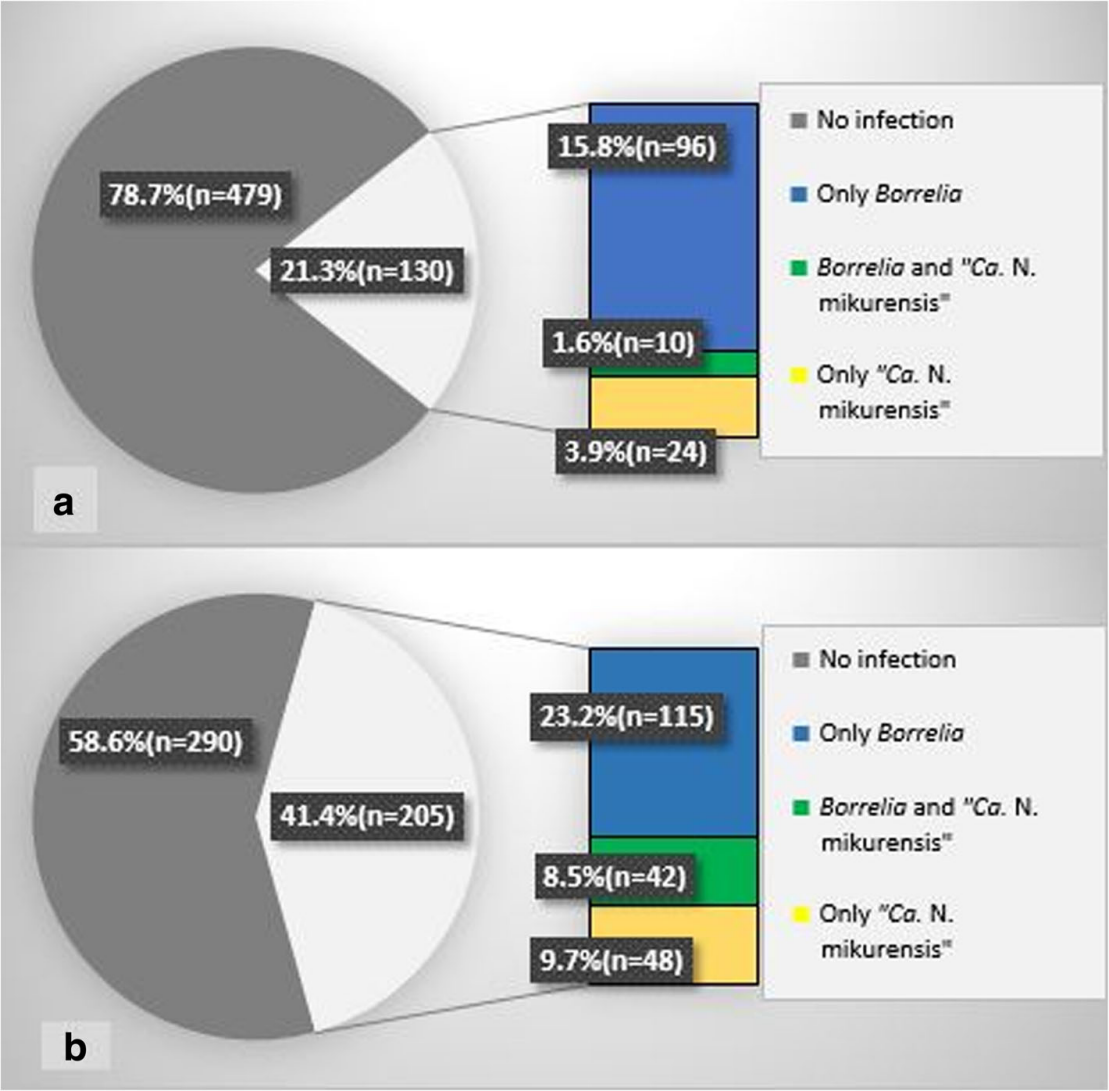
**a** Of 609 samples from *I. ricinus* ticks collected from dogs and cats in northern Norway, 21.3% were infected with *Borrelia* and/or “*Ca.* N. mikurensis”. **b** Of 495 samples from *I. ricinus* ticks collected by flagging in northern Norway, 41.4% were infected with *Borrelia* and/or “*Ca.* N. mikurensis”

Of the “*Ca.* N. mikurensis”-positive ticks from dogs and cats, 29.4% (10/34) had a simultaneous infection with *B. burgdorferi* (*s.l.*). Of the total 609 ticks collected from dogs and cats the proportion of coinfected was 1.6% (10/609, Fig. 2a).

### Ticks collected by flagging

From the *I. ricinus* ticks collected by flagging in Brønnøy 2011 [13], a total of 495 samples were analyzed for “*Ca.* N. mikurensis” by real-time PCR. Most of the ticks were nymphs, 64.4% (318/495), followed by adult males, 16.0% (79/495), adult females, 13.3% (66/495) and larvae 6.5% (32/495). The total proportion of “*Ca.* N. mikurensis”-positive ticks collected by flagging amounted to 18.2% (90/495).

There was no significant difference (Chi-square test*: χ*^2^ = 7.032, *df* = 3, *P* = 0.071) in the proportion of “*Ca.* N. mikurensis” positive ticks collected from vegetation between different life stages and sexes. Of the adult female ticks, 19.7% (13/66) were positive for “*Ca.* N. mikurensis”, of the adult male ticks 13.9% (11/79) and of the nymphs 20.4% (65/318). Of the samples derived from larvae, one sample (3.1%; 1/32) was positive for “*Ca.* N. mikurensis” in the real-time PCR assay, but it could not be confirmed by sequencing (Table 4).

Of the total 495 ticks collected by flagging from the vegetation in Brønnøy, 8.5% (42/495) had a co-infection with both *B. burgdorferi* (*s.l.*). and “*Ca.* N. mikurensis” (Fig. 2b). Of these ticks from the vegetation, 31.7% (157/495) had earlier been found to be positive for *B. burgdorferi* (*s.l.*) [13]. Among the adult ticks, infection with both pathogens was more common than single infection; 84.6% (11/13) of “*Ca.* N. mikurensis”-positive female ticks had a simultaneous *B. burgdorferi* (*s.l.*) infection, while 15.4% (2 of 13) did not. For male ticks, the corresponding proportion was 81.8% (9/11) coinfected and 18.2% (2/11) with only a “*Ca.* N. mikurensis” infection.

## Discussion

The purpose of this study was to investigate whether “*Ca*. N. mikurensis” infection occurs in one of the world’s northernmost populations of *I. ricinus* ticks, collected in the area surrounding the Arctic Circle in northern Norway. The study has shown that “*Ca.* N. mikurensis” is found in ticks from northern Norway and that the prevalence is higher in ticks collected from the vegetation compared to ticks that have fed from dogs and cats. The study has also shown that co-infection with *B. burgdorferi* (*s.l.*) and “*Ca.* N. mikurensis” occurs and that it is more common in ticks collected from the vegetation.

In the material that came from dogs and cats, the “*Ca.* N. mikurensis” prevalence was significantly higher in ticks collected in Brønnøy compared to ticks from the more northern districts, as has been shown for the *Borrelia* prevalence in a previous study [14]. One possible explanation is that there is no established population of *I. ricinus* ticks north of 65°N latitude, and that these ticks are believed to have been spread recently with migratory birds [13]. But even in the Brønnøy area, the “*Ca.* N. mikurensis” prevalence is higher in ticks collected from the vegetation than in those collected from dogs and cats. The two separate groups differ in terms of the distribution between life stages and sex, which may be a confounding factor, but it is still hard to determine a single reason that completely explains the difference. One possible explanation may be that the number of *Borrelia* bacteria in ticks is influenced by the duration of tick feeding [20]. Ticks that had been feeding longer than 36 h had a significantly lower number of *Borrelia* bacteria compared to ticks with shorter duration of feeding. A possible reason for this is that the *Borrelia* pathogens migrate from the tick’s gut to its salivary glands and thereafter the pathogen is transferred from the tick to its host. We do not know if the same applies to “*Ca.* N. mikurensis”, but if it does it could explain the difference in prevalence between unfed ticks collected from the vegetation compared to fed ticks from dogs and cats. Previous studies from Europe [6, 21] indicate that the prevalence of “*Ca.* N. mikurensis” shows a strong seasonal variation. The two examined tick materials were collected at different times (July to October 2010, June to October 2011 and April to November 2011, respectively). This may be another explanation for the difference in prevalence. In any case, the different collection periods make it somewhat harder to draw firm conclusions about the reasons for a higher “*Ca.* N. mikurensis” prevalence in the ticks collected from the vegetation.

The prevalence of “*Ca.* N. mikurensis”-positive samples differed slightly between the different tick stages, with a somewhat lower proportion of positive samples from adult ticks, but the differences were relatively small. The significant difference found between life stages in the tick material collected from dogs and cats was based on a low number of ticks, and no firm conclusions can be drawn. A previous study from Sweden [22] indicates that the tick’s life stage does not seem to affect the infection rate of “*Ca.* N. mikurensis”, while a study from the Czech Republic [9] shows that “*Ca.* N. mikurensis” infection is twice as common in nymphs removed from humans compared to adult ticks.

Among the ticks collected from the vegetation in Brønnøy, one larva was positive for “*Ca*. N. mikurensis” by real-time PCR assay. A positive finding in a larva indicates that “*Ca*. N. mikurensis” may be transovarially transmitted or that the larva was interrupted during feeding on an infected host. Although unlikely, we cannot exclude the possibility that this tick specimen was incorrectly classified as a larva before analysis. However, our result remains equivocal because “*Ca*. N. mikurensis” infection in the tick could not be confirmed by sequencing.

The proportion of ticks that had either an infection with *B. burgdorferi* (*s.l.*) or “*Ca.* N. mikurensis” or both differed between the ticks collected from dogs and cats and the ticks collected by flagging. A previous study performed on *I. ricinus* ticks collected in different parts of the Netherlands [21] has shown that a co-infection with “*Ca.* N. mikurensis” and *B. afzelii* occur significantly more than random; in this study, a co-infection of “*Ca.* N. mikurensis” and *B. burgdorferi* (*s.l.*) was common. In a study [22] investigating the relationship between “*Ca.* N. mikurensis” and other simultaneous infections in ticks, it is proposed that a co-infection is probably due to that the original rodent host has several infections at the same time and not that the tick would have different infections from more than one host animal. There are studies suggesting that humans infected with both *Borrelia* and *Anaplasma phagocytophilum* can have a risk for more severe disease [23, 24]. Possibly, the same could apply to a co-infection with *B. burgdorferi* (*s.l.*) and “*Ca.* N. mikurensis”. However, other studies have shown contradictory results [25].

A large number of samples has been analysed in this study and it is likely that the results are representative for ticks in the examined area in northern Norway. A possible limitation in the study is that all analyses have been conducted on single replicates. Analyses of sample duplicates or triplicates would have increased the certainty of the results. However, this was compensated for by the fact that all samples have undergone two consecutive tests, both real-time PCR and a nested PCR.

## Conclusions

The present study has shown that “*Ca.* N. mikurensis” infection is present in up to 18.2% of *I. ricinus* ticks in different parts of northern Norway. The result is interesting since “*Ca.* N. mikurensis” can be transmitted to humans after a tick-bite and can then cause severe disease in immunosuppressed persons. Physicians who meet persons with unexplained fever, skin rashes and thromboembolic events should have neoehrlichiosis in mind and ask for previous tick-bites, especially in cases of underlying autoimmune, haemolytic diseases or immunosuppressive treatment.
